# The association of conspiracy beliefs and the uptake of COVID-19 vaccination: a cross-sectional study

**DOI:** 10.1186/s12889-023-15603-0

**Published:** 2023-04-11

**Authors:** Kinga Kowalska-Duplaga, Mariusz Duplaga

**Affiliations:** Department of Health Promotion and e-Health, Institute of Public Health, Faculty of Health Sciences, Jagiellonian University Medical College, Skawinska Str. 8, Krakow, 30-611 Poland

**Keywords:** COVID-19 vaccination, Generic conspiracist beliefs, Vaccine conspiracy beliefs, COVID-19-related conspiracy beliefs, Vaccine hesitancy, Vaccine uptake, Vaccine acceptance, Political partisanship

## Abstract

The COVID-19 pandemic revealed that health denialism might be an important determinant of adherence to preventive measures during epidemic challenges. Conspiracy beliefs seem to be one of the most visible manifestations of denialism in society. Despite intensive efforts to promote COVID-19 vaccinations, the number of citizens reluctant to get vaccinated was very large in many countries. The main aim of this study was the analysis of the association between the acceptance of the COVID-19 vaccination and conspiracy beliefs among adult Internet users in Poland. The analysis was based on data from a survey performed on a sample of 2008 respondents in October 2021. Uni- and multivariable logistic regression models were applied to evaluate the association between attitudes towards COVID-19 vaccination and generic conspiracist, vaccine-conspiracy, and COVID-19-related conspiracy beliefs. In the multivariable model, the effect of conspiracy beliefs was adjusted for the level of vaccine hesitancy, future anxiety, political sympathies, and socio-demographic variables. Univariate regression models showed that COVID-19 vaccination acceptance is significantly lower among respondents with higher levels of all three types of conspiracy beliefs. In the multivariable model, the effect of COVID-19-related and vaccine conspiracy beliefs, but not generic conspiracist beliefs, was maintained after adjusting for vaccine hesitancy. We conclude that conspiracy beliefs should be treated as a potential indicator of lower adherence to preventive measures during epidemic challenges. The respondents revealing a high level of conspirational thinking are a potential group for intensified actions which employ health educational and motivational interventions.

**Supplementary Information:**

The online version contains supplementary material available at 10.1186/s12889-023-15603-0.

## Background

This study is focused on the importance of conspiracy beliefs in shaping the attitudes toward vaccination against COVID-19. It appears that the effect of such beliefs for public health became particularly evident during the COVID-19 pandemic. We have undertaken the challenge of assessing the role of three types of conspiracy beliefs (generic, COVID-19-related, and vaccine-related) in one model after adjusting, apart from socio-demographic variables, for other factors implicated in influencing vaccination decisions, such as health literacy, vaccine hesitancy, political sympathies, and anxiety level.

The general attitude of questioning scientific reasoning and rejecting evidence which to date has been irrefutable is typical of denialism. This term, used initially in psychology to indicate persons who deny reality to avoid facing an uncomfortable truth became popular in other contexts because of the prevalence of radical ideas based on the rejection of basic facts and the scientific consensus [1]. In their blog, Hoofnagle and Hoofnagle described denialism as the employment of rhetorical facts to give the appearance of argument or legitimate debate when, in actuality, there is none [2]. In 2009, Diethelm & McKee argued that public health specialists should be aware of denialism and able to recognize and react to it [3]. The characteristic elements of denialism include the use of fake experts; selectivity, meaning the recognition of isolated papers which challenge the dominant consensus; the creation of impossible research expectations; the use of misrepresentation and logical fallacies; and the identification of conspiracies [3].

Denialism is inherently associated with conspiratorial thinking, and identifying conspiracies is one of the key elements of denialism. Conspiracy theories reject the standard explanation of an event and attribute it to covert groups or organizations intending to carry out secret plots. Conspiracy theories are not unusual in the world of politics, but they are now very common in other areas, including science and health. It is also well-known that belief in political conspiracies is positively associated with accepting medical conspiracies [4].

An individual’s inclination to support theories assigning the responsibility for important societal events or phenomena to persons or groups acting with ill intentions is called a conspiracist mentality [5]. Conspiracist beliefs may lead to higher engagement in political activities to expose specific conspiracies or, if it is believed that the group behind the conspiracy is too powerful, to political passivity [6]. Conspiracy mentality and conspiracy beliefs may be important predictors of unfavorable health behaviors, e.g., not adhering to medical recommendations [7] or not accepting vaccination [8, 9]. According to Oliver & Wood, at least 49% of people in modern societies accept at least one medical conspiracy, and as many as 18% believe three or more such theories [8].

The role of conspiracy theories in shaping attitudes toward vaccinations has frequently been addressed. According to Kata et al., conspiracy theories are typical elements of antivaccination movements [9]. Jolley and Douglas reported that anti-vaccine conspiracy beliefs were negatively associated with vaccination intentions [10]. Furthermore, exposure to information supporting anti-vaccine conspiracy theories was associated with diminished intention to vaccinate [10], and Chen et al. confirmed that exposure to anti-vaccine conspiracy theories leads to less favorable attitudes toward the HPV vaccine in Chinese young adults [11].

The COVID-19 pandemic triggered an increased interest in the role of conspiracy theories in attitudes toward the vaccines developed in response to the epidemic threat [12]. Romer and Jamieson, based on two surveys performed in the early phase of the COVID-19 pandemic in the USA, reported that belief in COVID-19 conspiracy theories was inversely associated with the perceived threat of the pandemic, adopting preventive measures (e.g., wearing a face mask), perceived safety of vaccination and intention to be vaccinated against COVID-19 [13]. According to the study by Yang et al., only conspiracy theories related to vaccination and not about COVID-19 significantly impacted the intention to take COVID-19 vaccination in Chinese respondents [14].

Apart from the negative effect of conspiracy theories on vaccine willingness or acceptance [15], it was also reported that they increased vaccine hesitancy or a generally negative attitude toward vaccines [16]. A systematic review conducted by van Mulukom et al. focused on the antecedents and consequences of COVID-19 conspiracy beliefs [17]. They found that such beliefs depended on many variables, including personality traits, socio-demographic factors, thinking styles and biases, group identity, trust in authorities, and social media use. The consequences of accepting conspiracy beliefs were also related to, apart from vaccination intentions and willingness to undertake preventive measures, pseudoscientific health practices, psychological well-being, and some misguided behaviors [17]. A systematic review of global COVID-19 acceptance of Shakeel et al., among many factors decreasing the acceptance of COVID-19 vaccination, indicated conspiracy theories relating infertility to such vaccination spread on social media [18]. In Poland, popular COVID-19-related conspiracy theories included the claims that the SARS-CoV-2 virus was a result of genetic manipulations, that it was on purpose released from the laboratory, and that news about the coronavirus was made up to spread panic and to achieve political aims [19].

However, not many studies have evaluated the role of different types of conspiracy beliefs in the same study sample. Among the studies addressing the relationship between conspiracy beliefs and attitudes toward vaccination, most of them applied tools that assessed general conspiracy beliefs [20, 21] or, in the case of COVID-19 vaccination, COVID-19-related conspiracy beliefs [22, 23].

Shapiro et al. proposed a scale measuring the level of conspiracy beliefs related to vaccination – the Vaccine Conspiracy Belief Scale (VCBS) [24]. They showed that the VCBS score is negatively associated with parents’ willingness to vaccinate their sons against human papillomavirus (HPV) after controlling for socio-demographic variables, knowledge about HPV, and healthcare provider recommendation [24]. Several other teams later used this scale in their research [25–28]. The association between vaccine conspiracy beliefs measured with the VCBS and COVID-19 vaccine hesitancy, acceptance, or uptake was evaluated recently in many countries and populations [29–34]. Recently, Caycho-Rodriguez et al. also developed the COVID-19 Vaccine Conspiracy Beliefs Scale (COVID-VCBS) [35].

Brotherton et al. developed the Generic Conspiracists Beliefs Scale enabling measurement of conspiracist ideation, understood as a monological belief system relying on a limited number of generic assumptions about conspirational activity worldwide [36]. Some authors applied it in their studies of the attitudes and uptake of the COVID-19 vaccine [33, 37, 38].

Zaleski defined future anxiety as a state of apprehension, uncertainty, fear, worry, and concern that unfavorable changes are probable in a more distant personal future [39]. Our earlier study showed that greater future anxiety is associated with more intense COVID-19-related conspiracy beliefs [40]. On the other hand, some authors observed that persons with higher future anxiety reveal a greater willingness to get vaccinated with the COVID-19 vaccine [41] and lower reluctance to receive a booster dose of the vaccine [42]. Others reported that future anxiety was positively associated with a willingness to remain vigilant and adhere to preventive measures during the COVID-19 pandemic [43]. In turn, Scandurra et al. reported that anxiety about the future was a mediator of the reduction of the level of protective behaviors among Italians with lower trust in governmental organizations [44].

Political sympathies were reported as one of the significant determinants of adhering to recommended preventive measures during the pandemic, including the willingness or acceptance to take the COVID-19 vaccine. In the Polish population, higher adherence to preventive measures was predicted by a lower level of COVID-19-related conspiracy beliefs and political views, as reflected by the party supported during the last parliamentary election before the pandemic [45]. The effect of political views was maintained even in the multivariable model of adherence after adjusting for the level of conspiracy beliefs. Respondents supporting an extreme right-wing party or those not participating in the election were significantly less likely to adhere to preventive measures than a supporter of the ruling conservative party [45]. A significant association between political partisanship and attitudes toward COVID-19 vaccination was also reported by other authors [46–50], and some have indicated that the effect of political views on attitudes toward COVID-19 vaccination could be mediated by a predilection to vaccine conspiracy theories [51].

The topic of conspiracy beliefs is frequently examined in the literature in the context of the COVID-19 pandemic and readiness to get vaccinated [12–14, 16]. However, there are still gaps in the research that should be addressed. With our study we are going to explain the interplay between various types of conspiracy beliefs and their influence on the uptake of COVID-19 vaccination. In the last two years, many studies have addressed the relationship between vaccination practices and specific types of conspiracy beliefs. However, the current literature does not clearly establish whether generalized, vaccine-related, and COVID-19-related conspiracy beliefs add independently to the refusal to get vaccinated against COVID-19. It is also not clear if some specific aspects are covered by vaccine and COVID-19 conspiracy beliefs in relation to generalized conspiracy beliefs. Another question that still unanswered is to what degree conspiracy beliefs of various types are covered by the construct of vaccine hesitancy. The association between vaccine hesitancy and conspiracy beliefs is obvious [52], but it is not fully explained if the latter have an additional effect on the refusing COVID-19 vaccination. Finally, we lack a complex model showing the effect of conspiracy beliefs on COVID-19 vaccination after adjusting for health literacy. The general expectation is that health literacy should be a protecting factor against the impact of misinformation, including conspiracy beliefs, accompanying the COVID-19 pandemic [53–56], but unexpectedly, analysis combining health literacy and conspiracy beliefs in common models is not frequently reported.

The main aim of our study was to assess the association between generic conspiracist beliefs, vaccine- and COVID-19-related conspiracy beliefs, and attitudes towards vaccination against COVID-19. The rationale for the study was related to relatively high prevalence of conspiracy beliefs in Poland as reported from the beginning of the COVID-19 pandemic [19]. Furthermore, the uptake of COVID-19 vaccination is among the lowest in Europe [57]. To our knowledge, we are the first team to use the Polish version of the Vaccines Conspiracy Beliefs Scale (PL-VCBS) to assess the respondents’ decision about COVID-19 vaccination; therefore, we are reporting the results of the scale validation. To evaluate if three types of conspiracy beliefs predict vaccination decisions, we developed a multivariable regression model adjusting for the vaccine hesitancy score (derived from the adult Vaccine Hesitancy Scale, PL-aVHS [58]). Available evidence confirms the association between vaccine hesitancy and conspiracy beliefs, as discussed earlier. However, we also wanted to check if there are additional effects of different conspiracy beliefs, beyond the impact of vaccine hesitancy, in deciding on COVID-19 vaccination. The role of conspiracy beliefs was also adjusted for socio-demographic factors, future anxiety, political sympathies, and the use of social media. We hypothesized that all three types of conspiracy beliefs are independent predictors of lower acceptance of getting COVID-19 vaccination, adjusted for the level of vaccine hesitancy. We also wanted to verify the hypothesis that there are significant differences in COVID-19 vaccination acceptance between supporters of various political parties. Finally, we hypothesized that future anxiety predicts COVID-19 vaccine uptake.

The analysis was conducted on the data originating from the survey in the sample of Polish Internet users. The use of the online survey technique was dictated by the fact that we planned to include in the analysis not only health literacy but also e-health literacy. Furthermore, social media were indicated as a vehicle of misinformation during the pandemic.

## Materials and methods

### Survey

The analysis reported in this paper was based on data from a computer-assisted web-based interviewing (CAWI) survey performed among a sample of 2189 respondents representing the population of Polish adult Internet users. The survey was carried out by Ogólnopolski Panel Badawczy, a company conducting public opinion and marketing research, in November 2021. The respondents were selected from the Ariadna Internet Panel maintained by the Company. Assuming that in 2021, at least 24,000,000 Polish citizens aged 18–74 accessed the Internet at least once weekly, at a confidence level of 0.95 and a fraction of 0.5, the sampling error was 2.1% for this population. Stratified proportional sampling ensured that the structure of the sample corresponded to the structure of the population of Internet users in Poland concerning the place of residence, gender, level of education, age, and Nomenclature of Territorial Units for Statistics (NUTS) 1 region. The research team obtained acceptance from the Bioethical Committee established at Jagiellonian University (Decision No 1072.6120.99.2020 issued on April 23, 2020). Respondents invited to participate in the study were provided with information about the study’s aims and procedures. They had to confirm their agreement to join the survey before obtaining access to the questionnaire.

### Questionnaire

The questionnaire applied in the study was composed of 95 items. A set of validated instruments were used in the survey: the Adult Vaccine Hesitancy Scale (PL-aVHS) [58], the Generic Conspiracist Beliefs Scale (GCBS)[36], the 6 item version of the European Health Literacy Survey Questionnaire (HLS-EU-Q6)[59], the e-Health Literacy Scale (eHEALS)[60], the Future Anxiety Scale [61], the Vaccine Conspiracy Beliefs Scale (VCBS)[24], the COVID-19-related Conspiracy Beliefs Scale (CCBS)[19], and a set of items asking about the COVID-19 vaccination status of the respondent, about opinions on the COVID-19 pandemic, the use of social media and duration of the Internet use, health behaviors, political sympathies, and socio-demographic variables.

### Measures

#### Adult vaccine hesitancy scale (PL-aVHS)

The Adult Vaccine Hesitancy Scale (Pl-aVHS) was adapted to Polish and assessed for validity and reliability in an earlier study [58]. The original aVHS scale developed by Akel et al., based on a tool designed for the assessment of opinions of parents about the vaccination of children, consisted of 10 items [62]. Confirmatory Factor Analysis (CFA) of the Polish version of the scale showed that both 9 and 10-item versions show satisfactory characteristics as a measuring instrument. The answers to the items included in the scale can be provided on a 5-item Likert scale, from decidedly agree to decidedly disagree, with corresponding scores from 1 to 5. The answers to items 5, 9, and 10 are scored in reverse order. The total score of the 10-item version of the PL-aVHS ranges from 10 to 50. Cronbach α coefficient was equal 0.931, Guttman half-split coefficient 0.948.

#### Six-Item European Health Literacy Questionnaire (HLS-EU-Q6)

Various versions of the HLS-EU were earlier applied in survey studies performed in Poland [63–65]. The shortest available version of the HLS-EU, consisting of six items, was applied in the survey reported here [59, 63]. Response options to the questionnaire’s item span from ‘very difficult’ to ‘very easy.’ They are then converted to numerical values from 1 to 4. Respondents can also provide the response, ‘difficult to say/not applicable.’ This response option is treated as a missing value. The total score is calculated as a sum of individual scores if the number of missing values is not greater than 1. A total score ≤2 indicates inadequate, from > 2 to 3 indicates problematic, and > 3 indicates sufficient health literacy. Cronbach α coefficient was equal to 0.854, and Guttman half-split coefficient was 0.846.

#### e-Health literacy scale (eHEALS)

eHEALS was introduced in 2006 by Norman and Skinner as an instrument measuring digital health literacy [66]. A Polish version of the scale was developed by Duplaga et al. in 2019 [60]. It consists of 8 items that can be assigned with responses from ‘decidedly disagree’ to ‘decidedly agree.’ The response options are converted to numerical values from 1 to 5. The eHEALS score may range from 8 to 40. Cronbach α coefficient was equal to 0.931, Guttman half-split coefficient was 0.951.

#### Five-item future anxiety scale (FAS5)

The Seven-item Future Anxiety Scale (FAS5) is the shortest version of the tool developed by Zaleski to assess future anxiety [39]. The name Dark Future Scale is also used for the 5-item tool assessing future anxiety [61]. Respondents can select a response option from the 7-item Likert scale from ‘I decidedly do not agree’ to ‘I decidedly agree.’ These responses are converted to corresponding values from 1 to 7. The resulting FAS score can range from 7 to 35. Cronbach α coefficient was equal to 0.902, and Guttman half-split coefficient was 0.938.

#### Generic conspiracist beliefs scale (GCBS)

The Generic Conspiracist Beliefs Scale (GCBS) was developed by Brotherton et al. to measure individual differences in generic conspiracist ideation [36]. The Polish version of the scale was developed in 2019 by Siwiak et al. [67]. Respondents can provide a response to every 15 items from ‘definitely not true’ to ‘definitely true’ with a neutral response in the middle. The response options are converted to values from 1 to 5. The total GCBS score can range from 15 to 75. Cronbach α coefficient was equal to 0.939, and Guttman half-split coefficient was 0.948.

#### COVID-19-related conspiracy beliefs scale (CCBS)

The scale encompasses three items asking about three common conspiracy theories on the origin and spread of the new coronavirus. The scale was earlier used in the survey performed during the initial phase of the COVID-19 pandemic in Poland [68]. The response to the items can be provided according to a 5-item Likert scale, from ‘decidedly disagree’ to ‘decidedly agree.’ The score reflecting COVID-19-related conspiracy beliefs is calculated as a sum of individual scores received after converting the response option to values from 1 to 5. The scale may range from 3 to 15. Cronbach α coefficient was equal to 0.753, and Guttman half-split coefficient was 0.698.

#### Vaccine conspiracy beliefs scale (PL-VCBS)

The 7-item VCBS was developed and validated by Shapiro et al. [24]. They reported that the scale has a single-factor structure. We received consent from Dr Gilla K. Shapiro on behalf of the team that developed the tool to proceed with the Polish adaptation. The procedure of cultural adaptation was based on WHO guidelines [69]. First, two native Polish speakers with medical education prepared a forward translation of the VCBS. The translators adhered to the main assumptions of WHO guidelines. The main aim was to provide rather a conceptual equivalent instead of a word-for-word translation. Furthermore, wording appropriate for Polish cultural contexts was targeted. The translators were instructed to avoid the use of professional language or jargon.

An expert panel comprising six members with various backgrounds and competencies, including pediatrics, internal medicine, public health, nutrition, sociology, and linguistics, was established to proceed with cultural adaptation. The Panel examined both translations and proposed the final version of the Polish translation by consensus. One original item was substituted with an item more relevant to the Polish context. It was agreed that the item: “The government is trying to cover up the link between vaccines and autism” is specific to the views spread in the USA. It was substituted with the item formulated: “Physicians promote unnecessary vaccination because they collude with pharmaceutical companies.” The Panel agreed that such an item is more relevant for views popular in Poland in antivaccination environments.

After establishing the Polish version of the scale, two independent backward translations were prepared by translators with English as the mother language. It was ensured that the translators did not know the original version of the scale. They had no professional background in medicine or public health. The back-translated versions of the scale were compared with the original English scale. Basically, no major discrepancies between the original and back-translated versions of the scale were found apart from one item, which was completely substituted with the Polish version.

After checking the back-translated versions of the scale, the pilot phase was initiated with the agreed Polish version. The piloting was conducted on 21 respondents representing diversified characteristics concerning gender, age, and education level. The characteristics of the pilot group was reported earlier [58]. The respondents were provided with paper questionnaires containing additional fields for feedback on key issues important for cognitive interviewing. They were asked to describe their thoughts when they selected the response option to the scale’s items and their motivation to select a response. The participants of the pilot phase were also encouraged to indicate those terms or expressions that were unclear or understandable. The feedback for the respondents during the cognitive interviewing was analyzed by the expert panel. Overall, the piloting did not reveal problems in understanding the items included in the Polish version of the scale. Final Polish version of the VCBS is provided in the Supplementary Information file (Additional File 1: Table S1).

### Statistical analysis

Statistical analysis was carried out with IBM SPSS Statistics 28 and IBM SPSS Amos 28 software. Descriptive statistics calculated for categorical variables used in the analysis included absolute and relative frequencies. For continuous numerical variables, means and standard deviations (SD) were established.

### Reliability assessment

Internal consistency of the PL-VCBS was assessed based on the Cronbach α coefficient. It was assumed that good internal consistency is shown by values between 0.7 and 0.9 and excellent internal consistency by values > 0.9. We also calculated a Guttman split-half coefficient, assuming that a value of at least 0.8 determines adequate internal consistency of the tool.

The test-retest reliability was used as the indicator of the temporal stability of the instrument. It was calculated based on the results of the scale filled by 50 respondents twice at the interval of 2 weeks. The mean and single-item interclass correlation coefficients (ICC) were assessed assuming a two-way mixed model [70]. We followed the guidelines taking that a mean ICCs value < 0.40 indicates poor, 0.40–0.59 fair, 0.60–0.74 good, and 0.75-1.00 excellent stability. The floor and ceiling effects were assessed based on the percentages of respondents who received a score of 7 and 49 points, respectively. The adequacy of the sample size in relation to the number of items was analyzed with the Kaiser-Meyer-Olkin test. It was expected that the test would reach at least a value of 0.7 to confirm the adequacy of the sample size [71]. The factorability of the data was assessed with Barlett’s test of sphericity.

Hypothesis testing was applied to analyze the construct validity of PL-VCBS. We assessed the correlation between PL-VCBS and GCBS scores. We have also checked if there is a correlation between PL-VCBS and HL score, expecting no significant correlation.

### Exploratory Factor Analysis

The latent variables responsible for the variance of the scale were analyzed with EFA factoring. We randomly split the initial survey data set into two data sets. EFA was performed on the first data set. We applied the maximum likelihood method to extract latent factors. Before initiating the EFA, we analyzed the communalities values, assuming they should not be less than 0.2 [72]. We applied the Kaiser criterion to extract factors assuming that the factor’s eigenvalue should be equal to at least 1.0. The principal factors were extracted based on varimax orthogonal rotation. Factor loading > 0.4 was treated as a stable value [73, 74]. We also assumed that cross-loading of items should not be meaningful (below 75%). Finally, we expected the retained factors toe responsible for above 50% of the total variance [75].

### Confirmatory Factor Analysis (CFA)

CFA was performed on the second data set obtained after the random splitting the initial survey data set. The confirmatory factor analysis (CFA) was performed assuming a single factor structure of the VCBS, as reported by the authors of the original tool[24] and also in line with the results of EFA we performed. The fit of the factorial model was assessed. The estimation method of maximum likelihood was applied in the CFA. The goodness-of-fit of the model was evaluated based on several fit coefficients: the normed fit index (NFI), the relative fit index (RFI), the Tucker and Lewis Index (TLI), the comparative fit index (CFI), the goodness-of-fit index (GFI), the adjusted GFI according to degrees of freedom (AGFI), the chi2-to-degrees-of-freedom ration (CDFR), and the root-means-square error of approximation (RMSEA). Expected values established for fit indexes according to the available literature were as follows: for NFI ≥ 0.90, for RFI > 0.90, for TLI ≥ 0.90, for CFI > 0.95, for GFI ≥ 0.85, for AGFI ≥ 0.80, for CDFR < 3.0, and RMSEA < 0.05 for good and 0.05–0.08 for acceptable fit [76–78]. It was assumed that at least five indexes should achieve reference levels to confirm the acceptable goodness-of-fit of the data to the factor structure.

### Regression modelling

The uni- and multivariable logistic regression models were developed for the variable reflecting the respondent’s COVID-19 vaccination status. The independent variables assessed in univariable regression models included: the level of vaccine hesitancy, generic conspiracist beliefs, vaccine conspiracy beliefs, COVID-19-related conspiracy beliefs, health and e-health literacies, the level of future anxiety, the use of social media, political support assessed by the voting decisions during the last parliamentary election and socio-demographic variables (age, gender, place of residence, education, vocational status, marital status, net monthly income per household member). Their inclusion in the regression modeling was dictated by a review of the literature and the study’s assumptions. The variable reflecting the use of social media was included in the analysis as it was reported earlier that users might be more susceptible to vaccine hesitancy and attitudes rejecting the need for COVID-19 vaccination [79]. Political debate during the pandemic in Poland also revealed substantial differences between supporters of various political parties in their attitudes toward vaccination [80].

Only the independent variables showing significant association in univariable regression models were included in the multivariable model. Before the model was developed, the multicollinearity was tested. None of the variables fulfilled the criteria of multicollinearity (VIF > 4, tolerance < 0.25).

The regression model was assessed with the Hosmer-Lemeshow test. The value of Nagelkerke R2 was also calculated. For the independent variables, the odds ratio (OR), the 95% confidential interval (95%CI), and the p-value were reported. A p-value < 0.05 was deemed to be significant.

## Results

### Characteristics of the study group

In the study samples, 51.21% (n = 1121) were women, 37.87% (n = 829) were inhabitants of rural areas, and 29.14% (n = 638) were inhabitants of urban areas with a population of at least 100,000. 26.14% (n = 763) of respondents had a university education. Detailed socio-demographic characteristics, the structure showing political sympathies, and the use of social media are shown in Table 1.This section may be divided by subheadings. It should provide a concise and precise description of the experimental results, their interpretation, as well as the experimental conclusions that can be drawn.

**Table 1:** Characteristics of the study group

Variable	Response options	%	n
Gender	female	51.21	1121
	male	48.79	1068
Place of residence	rural	37.87	829
	urban below 20,000 inhabitants	12.84	281
	urban 20,000-100,000 inhabitants	20.15	441
	urban 100,000-500,000 inhabitants	17.31	379
	urban above 500,000 inhabitants	11.83	259
Education	lower than secondary	38.19	836
	secondary or post-sec. not university	35.68	781
	university Bachelors	7.04	154
	university Masters	19.10	418
Income	not more than 1501 PLN	15.76	345
	1501–3000 PLN	36.32	795
	more than 3000 PLN	26.54	581
	refusal to respond	21.38	468
Marital status	married	53.86	1179
	partnered	12.88	282
	single	22.70	497
	divorced, separated, or widowed	10.55	231
Vocational status	public or private sector employee	50.43	1104
	self-employed or farmer	7.26	159
	retired or on a disability pension	21.06	461
	high school or University student	4.80	105
	vocationally passive incl. unemployed	9.82	215
	a part-time job or other	6.62	145
Political support	Law and Justice (ruling party)	26.86	588
	Confederation	6.26	137
	Civic Coalition and allies	26.13	572
	Polish People’s Party	5.80	127
	Democratic Left Alliance	12.11	265
	other	1.69	37
	didn’t participate in the election	21.15	463
Use of social media	no	6.44	141
	yes	93.56	2048
COVID-19 vaccination status	not vaccinated and not going to get vaccinated	26.50	573
	vaccinated or in course of vaccination or decided to get vaccinated	73.50	1989

Abbreviations: PLN – Polish zloty.

Descriptive statistics of continuous variables reflecting age and scores in the study samples used in the analysis are presented in Table 2.

**Table 2:** Descriptive statistics of continuous numerical variables

Variable	n	Mean	SD	Range
Age	2189	44.1	15.25	18–75
VHS	2189	24.71	8.29	10–50
VCBS	2189	26.4	10.63	7–49
GCBS	2189	45.19	12.25	15–75
CCSB	2189	12.67	4.13	3–21
FAS	2189	23.75	6.33	5–35
eHEALS	2189	28.91	5.17	8–40
HL*	1822	2.76	0.56	1–4

Abbreviations: SD – standard deviation, VHS – vaccine hesitancy score, VCBS – vaccine conspiracy belief score, GCBS – generic conspiracist beliefs score, CCBS – COVID-19-related conspiracy belief score, FAS – future anxiety scale, HL – health literacy, eHEALS – e-health literacy scale, * - the number of respondents for which health literacy score could be calculated (not more than one missing response or “don’t know/not applicable” response)

### Internal Consistency of PL-VCBS

The floor effect was equal to 5.6%, and the ceiling effect to 1.8%. Cronbach α coefficient was equal to 0.964, and Guttman half-split coefficient was 0.951. Both coefficients showed excellent internal consistency of the instrument. The correlation of individual items to the total score was between 0.792 and 0.905 (Additional File 1: Table S2) The Cronbach’s α coefficients calculated after removing individual items were lower for all items (Additional File 1: Table S2). The mean ICCs of the PL-VCBS for a two-week interval was 0.826 (95%CI: 0.691–0.902), confirming excellent stability. The single item ICC was 0.703 (95%CI: 0.528–0.821).

### Exploratory Factor Analysis of the PL-VCBS

The Kaiser-Meyer-Olkin test was 0.954, confirming an adequate sample size to carry out the EFA. The correlation matrix factorability was confirmed by Barlett’s sphericity test (chi2 = 7625.74, p < 0.001). The communality scores were between 0.630 and 0.833 (Additional File 1: Table S2). The EFA revealed a one-factor model (Additional File 1: Table S3, Figure S1). One factor explained 81.87% of the variance. Its initial eigenvalue was 5.74, and after extraction, 5.53. After extraction, one factor explained 78.98% of the variance. Factor’s loadings of individual items were between 0.804 and 0.928 (Additional File 1: Table S4).

### Confirmatory Factor Analysis of the PL-VCBS

**Fig. 1:**
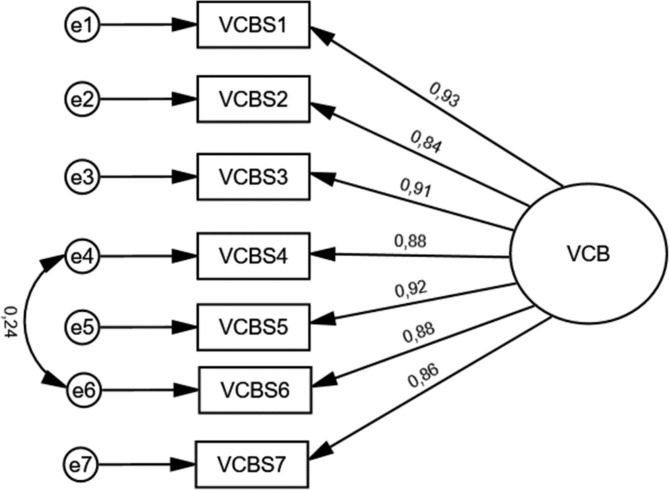
The measurement model for the PL-VCBS.

The measurement model for the VCBS is shown in Fig. 1. CFA of the PLVCBS, assuming a one-factor structure of the tool, showed acceptable fitting. NFI, GFI, AGFI, CFI RFI, and TLI had values showing good performance (Table 3). RMSEA (90% CI) was equal to 0.063 (0.050–0.077).

**Table 3:** The fitting results of the one-factor model of the Polish version of the Vaccine Conspiracy Beliefs Scale (PL-VCBS).

Indexes	Threshold levels of indexes	Fitting of one-factor model
CDFR	< 2.0 (p > 0.05)	5.938 (p < 0.001)
NFI	Acceptable: ≥0.90 to < 0.95, good: ≥0.95	0.992
GFI	Acceptable: ≥0.90 to < 0.95, good: ≥0.95	0.983
AGFI	Acceptable: ≥0.90 to < 0.95, good: ≥0.95	0.963
CFI	Acceptable: 0.90–0.95, good: ≥0.95	0.994
TLI	Acceptable: 0.90–0.95, good: >0.95	0.990
RFI	Acceptable: ≥0.90 to < 0.95, good: ≥0.95	0.987
RMSEA (90%CI)	Acceptable: <0.08 to 0.05, good: <0.05	0.063 (0.50-0.077)

Abbreviations: CDFR – the chi2-to-degree-of-freedom ratio, NFI – normed fit index, GFI – Goodness of Fit index, AGFI – adjusted goodness of fit index, CFI – comparative fit index, TLI – Tucker-Lewis fit index, RFI – relative fit index, RMSEA - the root-means-square error of approximation, 90%CI – 90% confidence interval.

### Univariable logistic regression modeling of COVID-19 vaccination

Simple regression models showed that older persons were more likely than younger (OR, 95%CI: 1.03, 1.03–1.04), inhabitants of great cities more likely than inhabitants of rural areas (1.66, 1.17–2.36), respondents with a university education were more likely than those with lower than secondary education (for university Bachelors vs. lower than secondary 1.55, 1.02–2.37, and university Masters vs. lower than secondary, 1.41, 1.07–1.86), persons with higher income were more likely than those with the lowest monthly net income per household member (1.55, 1.18–2.05 for income 1501–3000 PLN and 2.15, 1.58–2.91 for income > 3000 PLN), and the self-employed and students were more likely than employees to get vaccinated against COVID-19 (1.59, 1.10–2.29 and 3.07, 2.02–4.69, respectively) (Table 4). Persons living in partnership and singles were less likely to undergo vaccination than married people (OR, 95%CI: 0.64, 0.48–0.86, and 0.64, 0.51–0.81, respectively).

Supporters of the far-right party were more than 60% less likely to get vaccinated than supporters of Law and Justice, the ruling party (OR, 95%CI: 0.39, 0.26–0.56). Also, persons not participating in the last parliamentary elections were less likely to accept vaccination. The supporters of opposition parties showed a significantly higher acceptance of COVID-19 vaccination than supporters of the ruling party. Those accessing social media were 50% less prone to get vaccinated (OR, 95%CI: 0.50, 0.31–0.79).

The acceptance of COVID-19 vaccination was significantly associated with the level of vaccine hesitancy (OR, 95%CI: 0.77, 0.75–0.79), generic conspiracist beliefs (0.94, 0.93–0.95), vaccine conspiracy beliefs (0.86, 0.85–0.88), and COVID-19-related conspiracy beliefs (0.78, 0.76–0.81). A higher likelihood of vaccination was also observed among persons with higher future anxiety (1.02, 1.01–1.03). Interestingly, neither health literacy (1.15, 0.95–1.39) nor digital health literacy (0.996, 0.98–1.02) were significantly associated with undergoing COVID-19 vaccination. Detailed results of univariable logistic regression are presented in Table 4.

**Table 4:** Simple logistic regression models of the acceptance of COVID-19 vaccination

Variable	Response options	OR (95%CI)	p-value
VHS		0.77 (0.75–0.79)	< 0.001
VCBS		0.86 (0.85–0.88)	< 0.001
GCBS		0.94 (0.93–0.95)	< 0.001
CCBS		0.78 (0.76–0.81)	< 0.001
HL		1.15 (0.95–1.39)	0.142
eHEALS		0.996 (0.98–1.02)	0.685
FAS		1.02 (1.01–1.03)	0.002
Age		1.03 (1.025–1.04)	< 0.001
Gender	female*		
	male	1.12 (0.93–1.36)	0.237
Place of residence	rural*		
	urban below 20,000 inhabitants	0.91 (0.67–1.23)	0.548
	urban below 20,000-100,000 inhabitants	0.95 (0.73–1.23)	0.687
	urban below 100,000-500,000 inhabitants	0.98 (0.74–1.29)	0.864
	urban below above 500,000 inhabitants	1.66 (1.17–2.36)	0.005
Education	lower than secondary*		
	secondary or post-sec., not university	1.02 (0.82–1.27)	0.847
	university Bachelors	1.55 (1.02–2.37)	0.041
	university Masters	1.41 (1.07–1.86)	0.016
Income	not more than 1501 PLN*		
	1501–3000 PLN	1.55 (1.18–2.05)	0.002
	more than 3000 PLN	2.15 (1.58–2.91)	< 0.001
	refusal to respond	1.24 (0.92–1.67)	0.164
Marital status	married*		
	partnered	0.64 (0.48–0.86)	0.003
	single	0.64 (0.51–0.81)	< 0.001
	divorced, separated, or widowed	1.00 (0.72–1.40)	0.99
Vocational status	public or private sector employee*		
	self-employed or farmer	1.59 (1.10–2.29)	0.013
	retired or on a disability pension	1.62 (0.99–2.64)	0.055
	high school or University student	3.07 (2.02–4.69)	< 0.001
	vocationally passive incl. unemployed	1.56 (0.90–2.70)	0.110
	a part-time job or other	1.02 (0.66–1.59)	0.927
Political support	Law and Justice (ruling party)*		
	Confederation	0.39 (0.26–0.56)	< 0.001
	Civic Coalition and allies	3.05 (2.24–4.14)	< 0.001
	Polish People’s Party	1.66 (1.04–2.65)	0.033
	Democratic Left Alliance	2.46 (1.68–3.62)	< 0.001
	Other	1.23 (0.57–2.69)	0.597
	Didn’t participate in the election	0.62 (0.48–0.80)	< 0.001
Use of social media	No*		
	Yes	0.50 (0.31–0.79)	0.003

Abbreviations: VHS – vaccine hesitancy score, VCBS – vaccine conspiracy beliefs score, GCBS – generic conspiracist beliefs score, CCBS – COVID-19-related conspiracy beliefs score, FAS – future anxiety score, PLN – Polish zloty, OR (95%CI) – odds ratio (95% confidence interval), *-reference category of variable.

### Multivariable logistic regression of COVID-19 vaccination

A multivariable logistic regression model showed that from the socio-demographic variables retained for the analysis, only older age and higher income were significant predictors of being vaccinated against COVID-19 (Table 5). Each year of age was associated with a 3% higher likelihood of receiving the vaccine. Persons with the highest net monthly income per household member were two times more likely to be vaccinated than those from the lowest income group (OR, 95%CI: 2.00, 1.30–3.07). A significant difference in being vaccinated was observed between the supporters of the main opposition party, Civic Coalition, and the ruling Law and Justice party (OR, 95%CI: 1.61, 1.07–2.43)

Greater future anxiety was associated with a higher likelihood of being vaccinated (OR, 95%CI: 1.03, 1.01–1.06). Higher levels of vaccine hesitancy, vaccine conspiracy beliefs, and COVID-19-related conspiracy beliefs were associated with significantly lower odds of getting vaccinated (OR, 95%CI: 0.83, 0.79–0.84, 0.94, 0.92–0.96, and 0.93, 0.88–0.97, respectively). In the multivariable model, the level of generic conspiracist beliefs did not maintain its effect (OR, 95%CI: 1.00, 0.98–1.01).

**Table 5:** Multivariable logistic regression model of the acceptance of COVID-19 vaccination (Hosmer-Lemeshow test chi^2^ = 27.436, df = 8, p < 0.001, Nagelkerke R^2^ = 0.563)

Variable	Response options	OR (95%CI)	p-value
VCBS		0.94 (0.92–0.96)	< 0.001
GCBS		1.00 (0.98–1.01)	0.722
CCBS		0.93 (0.88–0.97)	0.001
VHS		0.82 (0.79–0.84)	< 0.001
FAS		1.03 (1.01–1.06)	0.006
Age		1.02 (1.01–1.03)	0.002
Place of residence	rural*		
	urban below 20,000 inhabitants	1.03 (0.68–1.56)	0.877
	urban below 20,000-100,000 inhabitants	0.81 (0.57–1.16)	0.249
	urban below 100,000-500,000 inhabitants	0.78 (0.53–1.16)	0.221
	urban below above 500,000 inhabitants	0.84 (0.51–1.37)	0.486
Education	lower than secondary*		
	secondary	0.96 (0.71–1.30)	0.798
	university Bachelors	1.24 (0.71–2.16)	0.444
	university Masters	1.36 (0.92–2.01)	0.126
Income	not more than 1500 PLN*		
	1501–3000 PLN	1.39 (0.95–2.03)	0.086
	more than 3000 PLN	2.00 (1.30–3.07)	0.001
	refusal to respond	1.32 (0.88–1.96)	0.176
Marital status	married*		
	partnered	0.84 (0.56–1.28)	0.426
	single	1.02 (0.73–1.44)	0.906
	divorced, separated, or widowed	0.86 (0.53–1.37)	0.523
Vocational status	public or private sector employee*		
	self-employed or farmer	0.97 (0.58–1.64)	0.919
	retired or on a disability pension	1.05 (0.65–1.70)	0.848
	high school or University student	1.19 (0.64–2.22)	0.581
	vocationally passive incl. unemployed	0.84 (0.55–1.29)	0.426
	a part-time job or other	0.91 (0.55–1.49)	0.709
Political support	Law and Justice (ruling party)*		
	Confederation	0.68 (0.40–1.13)	0.139
	Civic Coalition and allies	1.61 (1.07–2.43)	0.022
	Polish People’s Party	1.71 (0.92–3.16)	0.088
	Democratic Left Alliance	1.33 (0.80–2.22)	0.275
	Other	1.81 (0.68–4.83)	0.234
	Didn’t participate in the election	0.83 (0.59–1.17)	0.277
Use of social media	No*		
	Yes	0.71 (0.38–1.32)	0.278

Abbreviations: VHS – vaccine hesitancy score, VCBS – vaccine conspiracy belief score, GCBS – generic conspiracist beliefs score, CCBS – COVID-19-related conspiracy beliefs score, FAS – future anxiety score, HL – health literacy, eHEALS – e-health literacy scale, PLN – Polish zloty, OR (95%CI) – odds ratio (95% confidence interval), *-reference category of variable.

## Discussion

CFA of the Polish version of the VCBS showed good fitting of the measurement model when the one-factor model of the scale was considered. Six of the eight applied indexes showed good fitting, one showed acceptable fitting (RMSEA), and only the CDMR was higher than accepted. However, as commented on by other authors, the CDMR usually reaches higher values in the case of numerous study samples [81, 82].

Simple logistic regression models showed that many of the variables considered independent were significantly associated with the uptake of the vaccination against COVID-19. Among socio-demographic factors, age, place of residence, education, marital status, vocational status, and income level were significantly associated with the variable reflecting vaccine uptake. Furthermore, significant differences were observed in comparisons between supporters of the ruling party and those who voted for other parties or did not participate in the elections. Users of social media were significantly less likely to get vaccinated. In turn, persons experiencing higher future anxiety were more likely to undergo vaccination. Respondents presenting higher vaccine hesitancy or one of three types of conspiracy beliefs (generic conspiracist, vaccine, or COVID-19-related) were less likely to undergo vaccination. Surprisingly, neither health literacy nor e-health literacy was significantly associated with vaccination status.

The multivariable regression model showed that only selected socio-demographic predictors retained a significant relationship with vaccination status. This was confirmed for age, and income level. Future anxiety retained an independent effect on vaccination uptake. Significant association with a lower likelihood of vaccination was seen for vaccine hesitancy and vaccine- and COVID-19-related conspiracy beliefs but not for generic conspiracist beliefs.

To our knowledge, it is the first study in which the effects of generic conspiracist beliefs, vaccine, and COVID-19-related conspiracy beliefs were examined in one model. The effect of generic conspiracist beliefs on COVID-19 vaccination vanished after including conspiracy beliefs specifically associated with COVID-19 or vaccination. It seems that the effect of generic conspiracist ideation is included in conspiracy beliefs focused on the origin of COVID-19 and the use of vaccines. Most authors applied only one type of instrument measuring conspiracy beliefs. Vaccine conspiracy beliefs were reported as a factor in increasing vaccine hesitancy or decreasing vaccine acceptance or uptake by several authors [11, 24, 26, 28].

From the beginning, the COVID-19 pandemic was associated with many conspiracy beliefs related to the origin of the new coronavirus, the routes of its dissemination, and the phenomena associated with the pandemic. This resulted in not only vaccines developed to prevent infection being targeted by pseudoscientific theories, but the disease itself also being prolific in raising conspiracy thinking. Many researchers analyzed either the effect of general vaccine conspiracy theories [29, 31, 32, 34, 83] or COVID-19-related conspiracy theories [22, 23] on the acceptance of developed vaccines. Finally, some authors developed a tool to assess conspiracy theories focusing on vaccines developed against COVID-19 [35].

Yang et al. evaluated the effect of both vaccine and COVID-19-related conspiracy beliefs [14]. Interestingly, they observed a significant association between vaccine conspiracy beliefs and intention to get vaccinated against COVID-19. Such an effect was not confirmed for conspiracy beliefs related to COVID-19.

In the multivariable model, we have included three scores of conspiracy beliefs and vaccine hesitancy. Earlier studies clearly showed vaccine conspiracy beliefs to be strongly associated with vaccine hesitancy [16, 17, 26]. Still, the independent effect of conspiracy beliefs on vaccination uptake was maintained after adjusting for the effect of vaccine hesitancy. It seems obvious that the effect of conspiracist thinking goes beyond the doubts included in the construct of vaccine hesitancy.

The role of future anxiety in influencing vaccination decisions seems rather complex. Our earlier study showed that future anxiety positively correlates with COVID-19-related conspiracy beliefs [19]. Some authors suggested that future anxiety may lead to pandemic-related fatigue, and this, in turn, leads to reduced engagement in protective behaviors [84]. However, simultaneously, future anxiety may be a driver of the attitudes and decisions directed toward safeguarding against the consequences of the pandemic [41–43].

We observed that neither health literacy nor digital health literacy is significantly associated with COVID-19 vaccination. This is an unexpected finding as many authors underlined the importance of developing health literacy in societies as a remedy against the spread and acceptance of disinformation accompanying the pandemic [53, 55, 85, 86]. Furthermore, it is commonly believed that health literacy should positively influence adherence to preventive measures related to the pandemic. Indeed, in the earlier study assessing the factors impacting compliance with a set of preventive measures (not including vaccination) recommended during the COVID-19 pandemic in Poland, both health literacy and e-health literacy were positively associated with adhering to such behaviors [45]. However, the analysis presented in this paper shows that COVID-19 vaccination eludes this effect of health and e-health literacy. To some extent, this may be related to the fact that vaccination against COVID-19 became a subject of national debate referring to arguments outside the health domain and depending on political identification [87–89].

There are reports suggesting that more intense users of social media are more prone to abstain from vaccination [90–93]. Indeed, the univariable regression model confirmed that social media users show lower uptake of COVID-19 vaccination than non-users. However, this effect vanished in the multivariable model, suggesting that social media may be one of the channels for inciting vaccine hesitancy and spreading the conspiracy theories that play the main role in lowering uptake.

The univariable model revealed significant differences between supporters of parties participating in the last parliamentary election in Poland and the ruling party. However, in the multivariable model, only the difference between the main opposition party and the ruling party for the COVID-19 vaccine uptake was preserved. The difference between the ruling party and the Confederation, an extreme right-wing party openly boycotting preventive measures, including vaccination, was not maintained. This may be related to the fact that supporters of the Confederation frequently also believe in extreme conspiracy theories. Some authors suggested that in the case of political partisanship, conspiracy beliefs may be a mediator of its effect on vaccination [51].

The analysis presented in this paper strengthens the view that misinformation in the form of conspiracy theories is one of the key factors, apart from vaccine hesitancy, decreasing adherence to recommended COVID-19 vaccination. It may be a valid argument that apart from interventions focused on the promotion of recommended vaccines, additional measures counteracting the effects of conspiracy beliefs should be implemented during and beyond the pandemic. Roozenbeck et al. developed, by analogy to the process of medical immunization, the concept of an ‘inoculation’ that is supposed to reduce susceptibility to misinformation across cultures [94]. Other authors later replicated this approach [95].

It also seems that the effect of conspiracy beliefs should be considered when interventions promoting COVID-19 vaccination are designed. The multivariable mode of COVID-19 vaccine acceptance developed in this study suggests that independent predictors include vaccine hesitancy, conspiracy beliefs, and political sympathies. All these factors have played a considerable role in increasing the resistance to vaccination during the pandemic. Among this trio, probably only the phenomenon of vaccine hesitancy is eligible for health promotion and education interventions. The ‘inoculation’ theory has been applied earlier in various contexts, e.g., in politics, but recently gained popularity as an intervention in contested science, misinformation, and conspiracy theories. It assumes that ‘therapeutic’ inoculation messages conveying weakened versions of persuasive challenges will protect the audience from misinformation [96].

### Practical implications

The combined model developed in our study showed that the readiness to get vaccinated against COVID-19 depends on factors stemming from socio-political antecedents. These factors include vaccine hesitancy, vaccine and COVID-19-related conspiracy beliefs, the level of future anxiety and political views. The main implication from this observation is that changing society’s attitudes towards COVID-19 vaccination, at least in Polish society, eludes traditional interventions undertaken in public health and health promotion. To a significant extent, it is also beyond the scope of health care professionals communicating with their patients. Doubts in the form of increased vaccine hesitancy may be a result of the misinformation flooding the media during the pandemic. Obviously, the prevalence of conspiracy beliefs is an important element of such misinformation. It is also obvious that in Poland the resistance to public health interventions recommended during the pandemic by the Government, became for some political parties, a tool of gaining popularity among the potential electorate [80]. Our findings should incite the search for new methods of health communication that would be able to overcome the resistance to both vaccination and, also to other preventive measures essential for stopping the pandemic, originating from social and political circumstances. The technique of “inoculation” addressed earlier could be one such approach. Seeking consensus among political opponents and appealing to their sense of responsibility could be another option. However, it is hardly possible in the current environment of overwhelming political division.

The results of our study to some extent replicate the findings from other countries. However, we must admit that the level of conspiracy beliefs during the pandemic was unexpectedly high in Poland [19]. This could be a symptom of increased susceptibility to misinformation, and if so, should prompt more intense activities to support the empowerment of individuals and society in relation to public health issues.

The countermeasures against misinformation, especially conspiracy beliefs, should be an important element of the preparedness toolkit for challenges related to emerging epidemic threats. Such countermeasures should include transparent information about the origins of new pathological strains to public opinion as well as justification of undertaken preventive measures. The tool validated in this study for the Polish audience, the Vaccine Conspiracy Beliefs Scale, may be used to screen attitudes in the general population toward vaccination and the anticipation of the effectiveness of planned vaccination programs. Finally, it may be useful that the traditional epidemiological approach to the surveillance of epidemic phenomena is enhanced with a non-standard approach, in this case, by surveying the level of conspiracy beliefs.

### Limitations

It is an observational study, and any potential reasoning about the causal relationship should be very cautious. Furthermore, we analyzed the data from the survey performed about 1.5 years after the beginning of the pandemic in Poland and about 11 months from when the vaccine against COVID-19 became available. Consequently, we could not analyze the time dynamics of the observed relationship.

The following limitation of the study is related to the type of survey technique. With the CAWI survey, we face an underrepresentation of the groups that suffer from the digital divide and other accompanying deprivation types. As regular use of the Internet in Poland is the lowest among the oldest strata of the population, we could not check if observed relationships are also valid in these strata. It may be particularly important, as the complications resulting from COVID-19 are decidedly more frequent in the older population and among persons with chronic medical conditions.

On the occasion of this study, we also report the results of the confirmatory factor analysis of the Polish version of VCBS. To our knowledge, it is the first scale assessing vaccine-related conspiracy beliefs available in Polish. We are aware that due to the exceptional intensity of misinformation accompanying the COVID-19 pandemic, the level of conspiracist beliefs could be higher than before the pandemic.

## Conclusions

Conspiracy beliefs exert an added effect to vaccine hesitancy on attitudes toward and uptake of vaccination against COVID-19. Furthermore, even if conspiracy beliefs are frequently associated with extreme political identification, they are independently associated with practices related to COVID-19 vaccination. Our study has also revealed that conspiracy beliefs related to vaccination and COVID-19 have an independent effect on the vaccination uptake of COVID-19 vaccination. We believe that apart from standard intervention promoting vaccinations addressed to various stakeholders, more specific interventions targeted at conspiracy beliefs should be considered during the pandemic, including the use of the ‘inoculation’ method.

## Electronic supplementary material

Below is the link to the electronic supplementary material.

Supplementary Material 1

## Data Availability

The datasets generated and analyzed during the current study are available in the ZENODO, repository, https://zenodo.org/record/7706787#.ZAzipnbMI3s.

## Abbreviations

AGFI: adjusted goodness of fit index, aVHS – adult Vaccine Hesitancy Scale
CDFR: the chi2-to-degree-of-freedom ratio
CFI: comparative fit index
COVID-19: Coronavirus Disease 2019
CCBS: COVID-19-related conspiracy beliefs score
eHEALS: e-Health Literacy Scale
eHL: e-health literacy scale
FAS: future anxiety score
GCBS: generic conspiracist beliefs score
GFI: Goodness of Fit index
HL: health literacy
ICC: interclass correlation coefficients
NFI: normed fit index
OR (95%CI): odds ratio (95% confidence interval)
PLN: Polish zloty
*PL-VCBS*: Polish version of Vaccine Conspiracy Beliefs Scale
RFI: relative fit index
TLI: Tucker-Lewis fit index
VHS: vaccine hesitancy score
VCBS: vaccine conspiracy beliefs score
