# Supplementary material for: The association of conspiracy beliefs and the uptake of COVID-19 vaccination: a cross-sectional study
Source: BMC Public Health. 2023 Apr 11;23:672. doi: 10.1186/s12889-023-15603-0 (PMC10088115; doi:10.1186/s12889-023-15603-0)
Supplement: Supplementary file 1 — Supplementary Material 1 [file 12889_2023_15603_MOESM1_ESM.docx]

**Table S1. English and Polish versions of Vaccine Conspiracy Beliefs Scale**

| Item abbreviation | English items | Polish version |
| --- | --- | --- |
| VCBS1 | Vaccine safety data is often fabricated. | Oficjalne informacje o bezpieczeństwie szczepień są często nieprawdziwe. |
| VCBS2 | Immunizing children is harmful and this fact is covered up. | Szczepienie dzieci jest szkodliwe i jest to ukrywane. |
| VCBS3 | Pharmaceutical companies cover up the dangers of vaccines. | Firmy farmaceutyczne ukrywają zagrożenia związane ze szczepieniami. |
| VCBS4 | People are deceived about vaccine efficacy. | Ludziom przekazuje się fałszywe informacje na temat skuteczności szczepień. |
| VCBS5 | Vaccine efficacy data is often fabricated. | Oficjalne informacje na temat skuteczności szczepionek są często nieprawdziwe. |
| VCBS6 | People are deceived about vaccine safety. | Ludziom przekazuje się fałszywe informacje na temat bezpieczeństwa szczepionek. |
| VCBS7 | The government is trying to cover up the link between vaccines and autism. | Lekarze propagują niepotrzebne szczepienia bo są w zmowie z firmami farmaceutycznymi. |

**Table S2. Means, item-to-total correlations, Cronbach’s alphas after removing specific items and initial communalities for the Polish version of VCBS**

| Item | Mean (standard deviation) | Mean after removing an Item | The Variance of the scale after removing an Item | Item-Factor correlation | Squared item-factor correlation | Cronbach α after removing an item | Initial commu-nalities |
| --- | --- | --- | --- | --- | --- | --- | --- |
| VCBS1 | 3.80 (1.65) | 22.41 | 83.199 | 0.887 | 0.802 | 0.958 | 0.802 |
| VCBS2 | 3.38 (1.65) | 22.83 | 85.629 | 0.792 | 0.630 | 0.965 | 0.630 |
| VCBS3 | 4.00 (1.69) | 22.21 | 82.971 | 0.871 | 0.765 | 0.959 | 0.765 |
| VCBS4 | 3.79 (1.69) | 22.42 | 81.943 | 0.905 | 0.827 | 0.957 | 0.827 |
| VCBS5 | 3.80 (1.63) | 22.41 | 83.055 | 0.905 | 0.833 | 0.957 | 0.833 |
| VCBS6 | 3.76 (1.69) | 22.45 | 81.998 | 0.903 | 0.822 | 0.957 | 0.822 |
| VCBS7 | 3.65 (1.69) | 22.54 | 83.445 | 0.856 | 0.738 | 0.960 | 0.738 |

**Table S3. Total variance explained by the two-factor latent structure of the scale.**

| Factor | Initial Eigenvalues | | | Sums of Squared Loading after Extraction | | |
| --- | --- | --- | --- | --- | --- | --- |
|  | Total | % of variance | Cumulated % of variance | Total | % of variance | Cumulated % of variance |
| 1 | 5.782 | 82.603 | 82.603 | 5.584 | 79.765 | 79.765 |
| 2 | 0.333 | 4.763 | 87.367 |  |  |  |
| 3 | 0.248 | 3.549 | 90.916 |  |  |  |
| 4 | 0.200 | 2.856 | 93.771 |  |  |  |
| 5 | 0.164 | 2.345 | 96.117 |  |  |  |
| 6 | 0.151 | 2.162 | 98.279 |  |  |  |
| 7 | 0.120 | 1.721 | 100.000 |  |  |  |

Figure S1. Scree plot


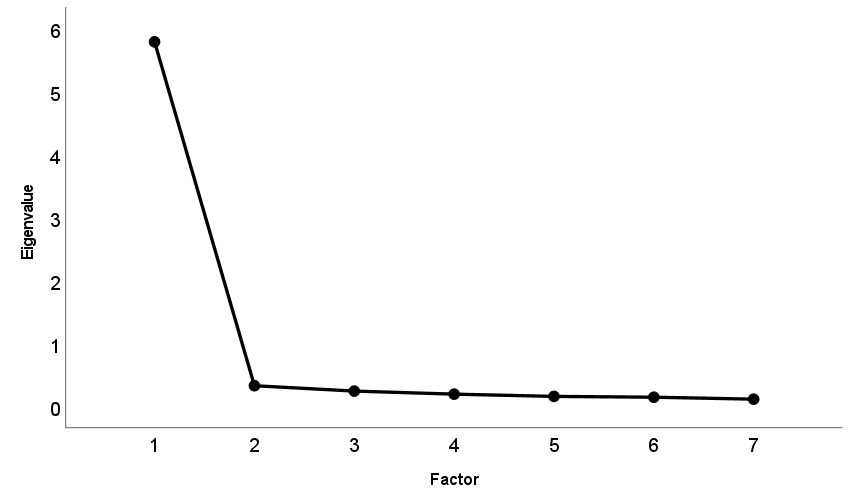


**Table S4. Factors loadings extracted with the maximum likelihood method**

| Factor | Factor loadings |
| --- | --- |
| VCBS1 | 0.908 |
| VCBS2 | 0.804 |
| VCBS3 | 0.887 |
| VCBS4 | 0.925 |
| VCBS5 | 0.928 |
| VCBS6 | 0.923 |
| VCBS7 | 0.870 |
